# Mapping the behavioral–affective network of physical activity and aggressive behavior among adolescents: a network and Bayesian graph analysis

**DOI:** 10.3389/fpsyg.2026.1779313

**Published:** 2026-08-05

**Authors:** Fang Yi, Ming Kiu Lee, Zhi Zhang

**Affiliations:** 1 College of Physical Education and Health, Guangxi Normal University, Guilin, China; 2 School of Physical Education, Jinan University, Guangzhou, China

**Keywords:** adolescent aggression, Bayesian network, conditional dependency, emotion regulation, expected influence, Gaussian graphical model, network intervention analysis, physical activity

## Abstract

**Background:**

Physical activity and aggression are important topics in adolescent psychology, but item-level links between physical activity (PA) and aggressive behavior (AGG) remain unclear.

**Methods:**

This cross-sectional study analyzed 1,276 Chinese secondary school students using standardized PA and AGG questionnaires. Regularized partial-correlation networks, expected-influence indices, network intervention response analysis, and Bayesian directed acyclic graphs were used to examine conditional dependency patterns.

**Results:**

The undirected network showed strong within-domain aggression dependencies and relatively sparse, weak PA-AGG cross-domain edges. Anger-related items (AGG14, AGG17, AGG15) and one physical-aggression item (AGG3) showed high expected influence. Network intervention response analysis indicated asymmetric activation patterns, and the Bayesian DAG placed anger-related items in upstream dependency positions.

**Conclusion:**

PA and AGG were relatively separate at the component level, while anger-related aggression items were central within the aggression system. Because data were cross-sectional, directed edges should be interpreted as exploratory conditional dependencies rather than causal or temporal pathways.

## 1 Introduction

Aggressive behavior in adolescence is increasingly recognized as a pervasive public health issue with serious implications for school adjustment, peer relationships, and psychological health (Gallardo-Nieto et al., 2021; Geoffroy et al., 2018). Adolescent aggression strongly predicts maladjustment in school and social domains, making it an important focus of developmental research (Nguyen-Louie et al., 2025). Aggression encompasses intentional actions or tendencies to harm others and is heterogeneous rather than unitary (Lawrence et al., 2025; Buss and Perry, 1992). This heterogeneity includes overt behavioral dimensions, such as physical and verbal aggression, as well as internal affective states, such as anger and hostility (Hood and Amir, 2018; Dewi and Kyranides, 2022; Anderson and Bushman, 2002; Benjamin Jr, 2015). From a developmental psychopathology perspective, failing to distinguish these components is problematic because they may have different etiologies and differential effects on long-term outcomes, including peer rejection and antisocial trajectories (Dodge et al., 2008; Watson et al., 2004). Therefore, treating aggressive behavior as a single construct may obscure the specific behavioral and emotional mechanisms that drive adverse developmental trajectories (Huesmann and Guerra, 1997; Vagos et al., 2025).

Conceptual models and growing evidence point to physical activity (PA) as a robust protective factor for such maladaptive behaviors (Rhodes and Nigg, 2011; Ouyang and Liu, 2023), operating possibly through its effects on regulation systems, executive functioning or the temporal channeling of aggressive tendencies into structured settings (Lubans et al., 2016; Martinez et al., 2024), From a self-determination perspective, the motivational quality of activity participation may also shape sustained engagement and related psychosocial outcomes (Deci and Ryan, 2000). However, the dynamic relationship between PA and aggression is still quite rudimentary. Primarily, studies in the area have considered both as two single constructs and used an aggregate scoring approach or latent variable modeling to represent linear relationships (Biddle and Asare, 2011). This streamlined approach inevitably obscures the heterogeneous associations at the group level; it overlooks the potentially differential inhibitory effects of different dimensions of participation (such as frequency, intensity, and duration) on specific aspects of aggressive behavior (such as physical violence and intrinsic hostility). Traditional analytical frameworks reduce these multidimensional structures to a single numerical value and may fail to capture complex nonlinear interactions between activity patterns and aggressive behavior. This limitation highlights that studying the macro-level relationships only between these two domains might neglect more important micro-level influence paths, such as dynamic interactions among components within the symptomatic layer.

The above methodological constraints are due to an incongruity of basic assumptions between traditional statistical methods and the complexities of PA aggression as psychological phenomena. In particular, correlational analyses and regression models generally presuppose that variables are mutually independent of one another and consider each such observed indicator as a passive mirror to an underlying latent entity rather than an active ingredient with causal agency (Borsboom and Cramer, 2013). Rather, physical activity and aggression form an interconnected set of systems where each component behaves such that it dynamically influences other components by means of both direct and indirect feedback loops among response or intervention units (Hofmann et al., 2016). For example, within the aggression system, the activation of angry affect could directly elicit hostile cognitions that may be involved in escalating physical aggressive acts, which in turn can serve to maintain or escalate angry tendencies through reinforcement of a person's aggressive self-concept (Cupaioli et al., 2021; Hofmann et al., 2016). Likewise, different features of PA participation, particularly exercise intensity, may be associated with distinct affective responses, suggesting that aggregate PA scores may obscure component-specific patterns (Ekkekakis et al., 2011; Rhodes and Kates, 2015). A further limitation of existing methods is that they cannot identify conditional associations between components while accounting for the status or activation level of other components in the network (Epskamp et al., 2018). Network analysis addresses this limitation by conceptualizing psychological constructs as systems of mutually interacting observable components and estimating the conditional association between each pair of components while accounting for all others (Borsboom and Cramer, 2013; Epskamp and Fried, 2018).

Recent applications of network modeling have shown promise in disentangling complex behavioral systems, although few studies have simultaneously examined items across the physical activity (PA) and aggression domains. The GGM can reveal patterns of conditional connectivity by identifying which behavioral and affective elements remain related after accounting for all other components (Epskamp and Fried, 2018; Chen et al., 2024). Beyond pairwise associations, centrality indices such as expected influence quantify the extent to which individual components are connected to the wider network (Robinaugh et al., 2016; Jones et al., 2021). Identifying influential nodes can help generate more precise hypotheses for behavioral intervention. Stability and accuracy checks also help determine whether estimated network patterns are robust rather than statistical noise (Epskamp et al., 2018). Applied to PA and aggression, this approach provides a detailed map of how adolescents' activity profiles and aggressive tendencies are conditionally interconnected.

Although network perspectives offer conceptual advantages, substantial empirical gaps remain regarding adolescent PA and aggression. To our knowledge, no study has systematically examined the joint item-level network structure of these two domains using complementary network approaches. Existing studies have generally modeled PA or aggression separately, leaving the structural positions of individual components and the bridging pathways between domains unclear. Evidence integrating undirected networks, centrality indices, network intervention response analysis, and Bayesian graph analysis is also limited. Addressing this gap may clarify central components of the PA-AGG network and provide an exploratory account of conditional dependency orientations.

Based on this theoretical and methodological foundation, this study employs a comprehensive network framework to construct a behavioral-affective map of physical activity and aggressive behavior among adolescents. Specifically, this investigation is guided by four objectives. First, we examine the conditional independence structure of PA and aggression at the item level, determining how specific activity dimensions (e.g., frequency and duration) are conditionally associated with distinct aggressive manifestations (e.g., anger and physical aggression) within a joint graphical model. Second, we identify the most central and influential nodes using expected-influence metrics, thereby locating components that may be especially relevant for sustaining the observed network structure. Third, we explore system responsiveness by analyzing how simulated changes in individual components are associated with overall network activation through network intervention response analysis (NiRA) (Zhang et al., 2018). Finally, to complement the undirected GGM, we investigate asymmetric probabilistic dependency patterns using Bayesian network modeling. Importantly, the Bayesian DAG is used here as an exploratory tool for conditional dependency orientation rather than as evidence of causal or temporal ordering.

By combining these complementary network approaches, the current study offers a component-level account of the PA-AGG system that extends conventional variable-centered work. It clarifies how specific physical-activity indicators are structurally related to symptoms of adolescent aggression and makes complex item-level patterns more transparent. These findings may inform the development of targeted hypotheses and future interventions designed to address aggressive behavior.

## 2 Methods

### 2.1 Participants and procedure

A total of 1,276 adolescents were included in the final analytic sample. Using a stratified sampling method, 1,500 upper-grade primary school students (Grades 4–6) were selected from the participating school. After students voluntarily provided informed consent/assent and guardian consent was obtained, they completed the online questionnaire through Wenjuanxing. A total of 1,500 questionnaires were distributed and returned. After excluding 224 invalid questionnaires, 1,276 valid questionnaires remained for analysis, yielding an effective response rate of 85.07%. Participation was voluntary, and students were informed that they could decline or withdraw without any academic or personal consequences. Written informed consent was obtained from legal guardians, and assent was obtained from the students before data collection. The inclusion criteria were as follows: (a) enrollment as an upper-grade primary school student (Grades 4–6) in the participating school, (b) ability to understand and complete the questionnaire independently, (c) provision of guardian consent and student assent, and (d) completion of the PAQ-A and BPAQ items required for the network analysis. The exclusion criteria were: (a) refusal to participate or withdrawal during data collection, (b) incomplete responses on PAQ-A or BPAQ items, or (c) clearly invalid response patterns identified during data screening. Because no demographic information was retained for students who refused participation or submitted invalid questionnaires, a formal comparison between participants and non-participants could not be conducted. The participating school was located in the Guangxi Zhuang Autonomous Region of China. Stratification was based on grade level, with students sampled from Grades 4, 5, and 6.

### 2.2 Measures

#### 2.2.1 Physical activity questionnaire for adolescents (PAQ-A)

Physical activity (PA) was measured using the Physical Activity Questionnaire for Adolescents (PAQ-A) (Li et al., 2015). The PAQ-A comprises nine items assessing moderate-to-vigorous physical activity during the previous seven days across typical daily settings, including physical education classes, leisure time, and evenings. Responses are scored on a 5-point Likert scale, with higher scores indicating higher levels of physical activity. Consistent with the intended use of the PAQ-A, the nine items were treated as indicators of a general physical-activity construct. All items were retained and analyzed as separate nodes in the network and Bayesian graph analyses.'

#### 2.2.2 Adolescent aggressive behavior scale

Aggressive behavior (AGG) was measured using the Buss–Perry Aggression Questionnaire (BPAQ) (Buss and Perry, 1992). The BPAQ comprises 20 items designed to assess aggressive tendencies, covering four theoretical dimensions: physical aggression, verbal aggression, anger, and hostility. Participants rate each item using a Likert scale, with higher scores indicating stronger aggressive tendencies.

### 2.3 Ethical consideration

This study was approved by the Ethics Committee of School of Guangxi Normal University (Ethics No. 25TYB400). Formal permission to conduct the study was obtained from the participating school and its administrators and teachers. Questionnaires were administered anonymously during school hours. No personally identifying information was collected. All information was kept confidential and was used for research purposes only. There was no financial or other material compensation for participation.

### 2.4 Data preprocessing

Before network estimation, the data were screened for completeness and validity. The primary analyses used the final analytic sample of adolescents who provided complete responses to all PAQ-A and BPAQ items. Thus, the item-level network matrix used for the GGM and Bayesian DAG contained no missing values on the PA and AGG nodes. When an individual had missing responses on any PAQ-A or BPAQ item, that case was excluded from the item-level network analysis by listwise deletion; no statistical imputation was performed. All PAQ-A and BPAQ items were treated as ordinal indicators. No additional reverse scoring was required because item coding and preliminary data screening had been completed before the network analyses.

### 2.5 Network analysis (Gaussian graphical model)

#### 2.5.1 Gaussian graphical model

We estimated an item-level Gaussian graphical model (GGM) to explore the conditional association structure between PA and AGG. In this model, nodes represented individual questionnaire items and edges represented regularized partial correlations between two items after conditioning on all other items in the network. To quantify cross-domain connectivity, we exported the final GGM edge-weight matrix and counted retained non-zero edges that connected PA nodes with AGG nodes. Because EBICglasso performs regularized model selection, retained edges were interpreted as conditional dependencies in the regularized network rather than as separate null-hypothesis significance tests for individual edges.

Because PAQ-A and BPAQ items are ordinal, network estimation was based on a Spearman rank-correlation matrix. The network was regularized and sparsified to improve interpretability and reduce the risk of retaining very small spurious edges. All network analyses were performed in R (version 4.5.2) using appropriate packages for network psychometrics.

#### 2.5.2 Centrality analysis

Node importance was assessed using expected influence (EI), which sums the signed edge weights connected to a node and is therefore suitable for networks that may contain both positive and negative associations. EI was selected as the primary centrality index because it preserves the direction of associations and is more informative than strength centrality when negative edges are present.

#### 2.5.3 Network stability

The robustness of network estimates was assessed based on nonparametric case-dropping bootstrap methods. A correlation-stability (CS) coefficient was computed to assess the stability of strength centrality as sample size decreased.' Expected influence was not included in the case-dropping bootstrap specified in the analysis script, so the Methods should not imply that its stability was directly evaluated.

#### 2.5.4 Network intervention analysis

We used network intervention analysis (NiRA) to investigate how potential perturbing manipulations of node activation would influence global network activity. For each node, two levels of the simulations were considered: an alleviating level (node's activation threshold lowered) and an aggravating level (node's activation threshold raised).

Amplitudes of changes in the global network activation under these conditions were assessed with respect to median reference. The median value of activation was employed to discern the typical state of a system and partly avoid impact from extremal values. Node-specific effects were estimated by bootstrap resampling procedures to determine its variability.

### 2.6 Bayesian graph analysis

To further examine asymmetric probabilistic dependency patterns among PA and AGG items, a Bayesian network was estimated using a hill-climbing search algorithm with the Bayesian Dirichlet equivalent (BDe) score criterion. The resulting DAG summarizes the orientation of conditional dependencies learned from the observed cross-sectional data.

The Bayesian DAG was used as an exploratory dependency-orientation method rather than as a causal discovery tool. Directed edges therefore indicate non-reciprocal probabilistic dependencies conditional on the other variables in the network and should not be interpreted as evidence of temporal precedence or causal effects.

### 2.7 Analytical scope and omitted-variable consideration

The primary network was pre-specified to include PAQ-A and BPAQ item nodes because the central research question concerned the component-level organization of physical activity and aggressive behavior. Other questionnaire modules were not included in the main network because adding conceptually heterogeneous variables would have changed the target network from a PA-AGG component network to a broader psychosocial network and reduced interpretability. However, this decision also means that the estimated edges are conditional only on the included PA and AGG items and not on all possible external correlates, such as emotion dysregulation, anxiety, depression, family functioning, or peer context. Consequently, the retained edges should be interpreted as conditional associations within the restricted node set, and potential omitted-variable confounding is addressed in the limitations.

## 3 Results

### 3.1 Descriptive statistics

Demographic characteristics of the sample regarding PA and AGG dimensions are presented in Table 1. Categorical data are expressed as frequencies and percentages, while PA and AGG scores are expressed as means and standard deviation.

**Table 1:** Demographic characteristics and descriptive statistics of physical activity (PA) and aggression (AGG) dimensions.

Variable	Category	Total	PA	AGG physical	AGG verbal	AGG anger	AGG hostility
Gender	Male	652 (51.10%)	3.01 (0.69)	2.24 (0.97)	1.96 (0.85)	1.72 (0.90)	2.33 (1.14)
	Female	624 (48.90%)	2.87 (0.64)	2.05 (0.87)	1.95 (0.81)	1.72 (0.91)	2.46 (1.13)
Only-child status	Only child	795 (62.30%)	2.94 (0.68)	2.13 (0.93)	1.95 (0.83)	1.70 (0.89)	2.39 (1.13)
	Not an only child	481 (37.70%)	2.95 (0.65)	2.18 (0.92)	1.97 (0.84)	1.74 (0.91)	2.39 (1.14)
Family location	Rural	14 (1.10%)	2.63 (1.06)	2.70 (1.26)	2.43 (1.07)	1.70 (1.06)	3.04 (1.35)
	Urban	1262 (98.90%)	2.94 (0.66)	2.14 (0.92)	1.95 (0.83)	1.72 (0.90)	2.38 (1.13)
Single-parent family	Yes	114 (8.93%)	2.92 (0.76)	2.39 (0.98)	2.10 (0.86)	1.84 (0.92)	2.64 (1.17)
	No	1162 (91.07%)	2.94 (0.66)	2.12 (0.92)	1.94 (0.83)	1.71 (0.90)	2.37 (1.13)
Age	–	11.22 (0.92)	2.94 (0.67)	2.15 (0.93)	1.95 (0.83)	1.72 (0.90)	2.39 (1.14)

Values for categorical demographic variables are presented as n (%). PA and AGG dimension scores are reported as Mean (SD). AGG dimensions include Physical, Verbal, Anger, and Hostility.

The sample was composed of 51.10% males and 48.90% females. On average, adolescents described themselves as moderately active (*M* = 2.94, SD = 0.67). PA scores are slightly higher for males than females, and females exhibit slightly higher H (hostility) aggression scale scores. Participants were aged 9–13 years: 0.71% were aged 9, 23.12% were aged 10, 37.85% were aged 11, 30.09% were aged 12, and 8.23% were aged 13. The sample included 527 Grade 4 students (41.30%), 385 Grade 5 students (30.17%), and 364 Grade 6 students (28.53%). In addition, 795 participants (62.30%) were only children, 114 (8.93%) were from single-parent families, 14 (1.10%) lived in rural areas, and 1,262 (98.90%) lived in urban areas.

For the entire sample, mean scores for physical aggression, verbal aggression, anger, and hostility were 2.15 (SD = 0.93), 1.95 (SD = 0.83), 1.72 (SD = 0.90), and 2.39 (SD = 1.14). Descriptive subgroup differences were generally small. Participants from single-parent families had higher mean physical-aggression and hostility scores, whereas mean PA scores were similar between groups. The rural subsample was very small (n = 14) and showed greater variability; therefore, these descriptive differences should be interpreted cautiously. Mean PA and AGG scores were similar between only-child and non-only-child groups.

### 3.2 Network structure of physical activity and aggressive behavior

**Figure 1:**
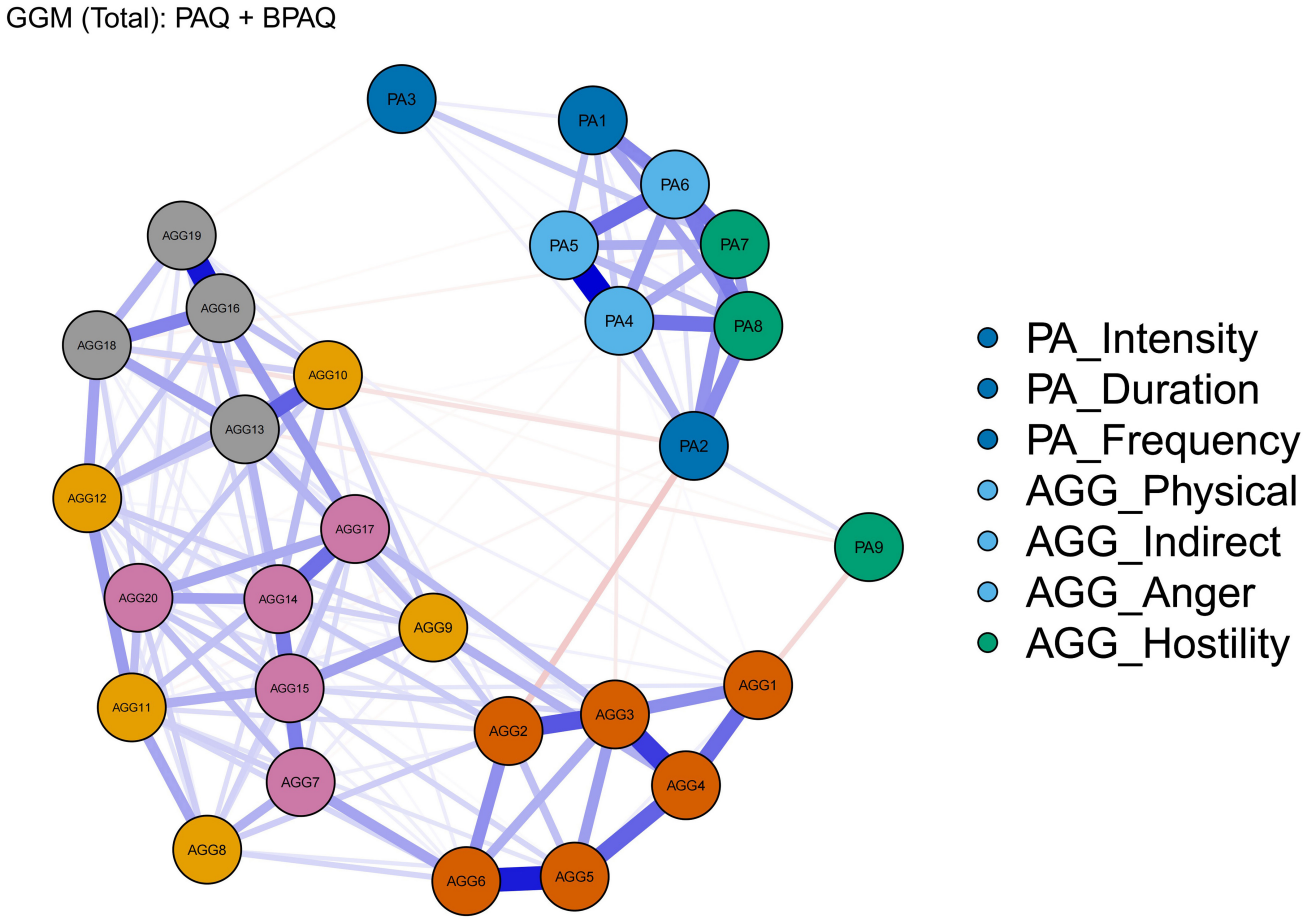
Network structure of physical activity (PA) and aggressive behavior (AGG) items. Blue edges indicate positive regularized partial correlations, red edges indicate negative regularized partial correlations, and edge thickness reflects the absolute magnitude of the estimated edge weight. The GGM is undirected; therefore, edges do not represent temporal or causal direction.

The item-level network structure of physical activity and aggressive behavior is shown in Figure 1. The inferred network had a clear modular structure, with PA and AGG items largely forming two relatively distinct clusters. Within-domain associations were generally stronger and mostly positive, whereas cross-domain PA-AGG connections were less frequent and weaker.

Activity frequency, duration, and intensity items were highly associated with one another within the PA cluster, indicating a coherent internal structure of physical-activity engagement. Several PA items occupied relatively central positions within the PA subnetwork, suggesting their relevance for maintaining internal connectivity among activity indicators.

The AGG items also showed a strong internal structure, clustering broadly in line with their theoretical subdimensions: physical aggression, verbal aggression, anger, and hostility. Strong within-domain associations were observed among items belonging to the same or closely related subdimensions, particularly among physical-aggression and anger-related items.

Cross-domain associations between PA and AGG were sparse and weak. A limited number of PA-AGG edges were negative, but these links were substantially weaker than the within-domain edges. Figure 1 shows that blue edges represent positive regularized partial correlations, red edges represent negative regularized partial correlations, and edge thickness reflects the absolute magnitude of the estimated partial correlation. Because the GGM is undirected, the edges in Figure 1 do not indicate temporal order or causal direction. In the final edge-weight matrix, 17 retained non-zero PA-AGG cross-domain edges were observed, with standardized partial correlations ranging from −0.072 to 0.006. Among these retained cross-domain edges, 15 were negative and 2 were positive. These values should be interpreted as small retained conditional dependencies in the regularized model rather than as separate null-hypothesis significance tests for each edge.

### 3.3 Expected influence and node importance

**Figure 2:**
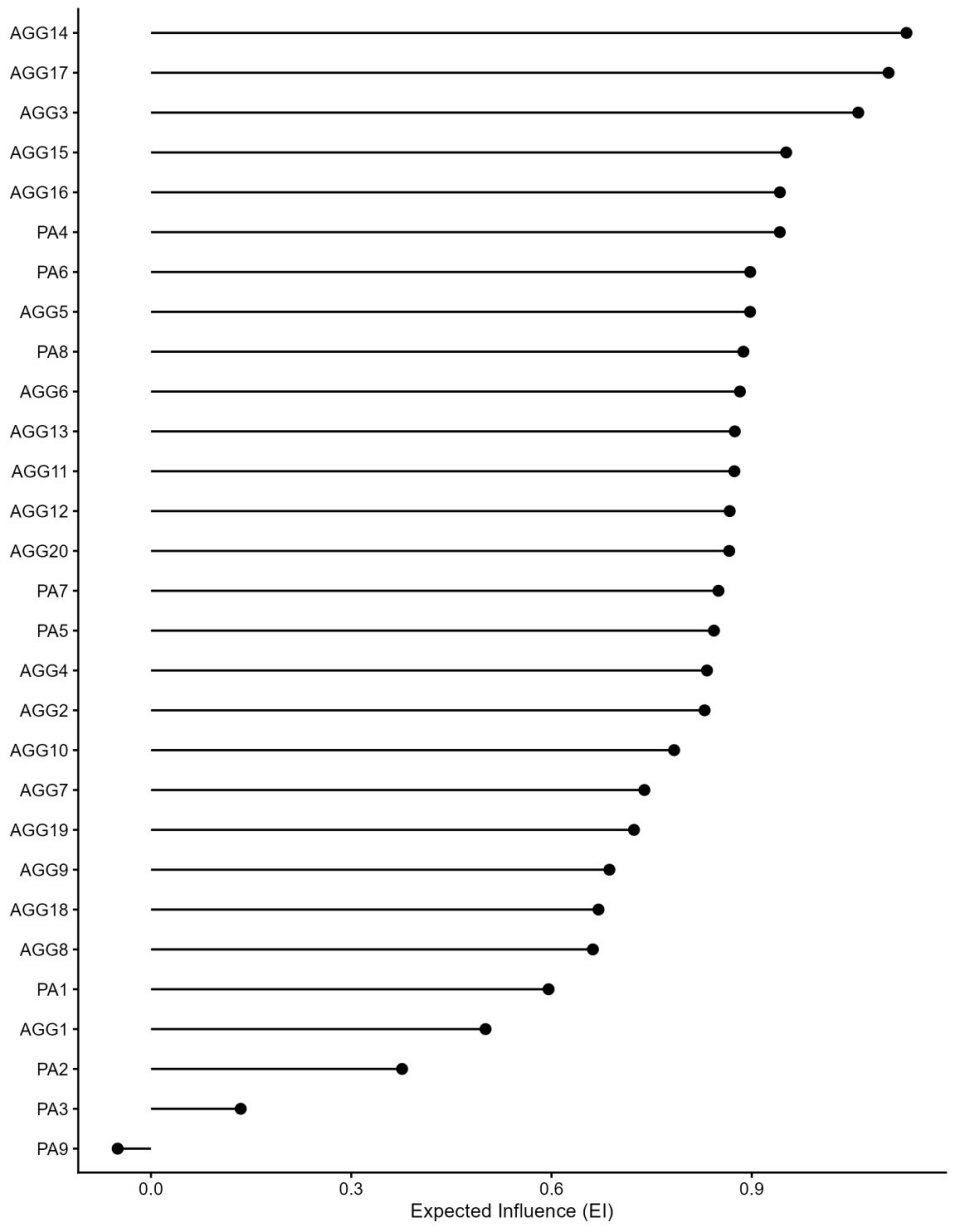
Expected influence (EI) of physical activity (PA) and aggressive behavior (AGG) items in the Gaussian graphical model.

Figure 2 presents the expected-influence (EI) values for all PA and AGG items in the Gaussian graphical model. EI was used to rank node importance by aggregating the signed values of all edges connected to each node.

Aggression items tended to dominate the higher EI values, suggesting that aggression-related nodes were more centrally located within the overall network. Notably, AGG14, AGG17, AGG3, and AGG15 showed high EI values, indicating broad conditional connectivity with other nodes in the network.

Among PA variables, PA4, PA6, and PA8 showed relatively higher EI than the other PA indicators, whereas PA1, PA2, PA3, and PA9 showed lower values. The correlation-stability coefficient for strength was CS = 0.75, indicating that strength rankings remained relatively stable under case-dropping bootstrap procedures.

### 3.4 Network intervention analysis (NiRA)

Applying network intervention response analysis, we examined how simulated changes in the activation threshold of each node were associated with overall network activation. Two parallel conditions were considered: an alleviating condition, in which a node threshold was shifted toward lower activation, and an aggravating condition, in which a node threshold was shifted toward higher activation.

**Figure 3:**
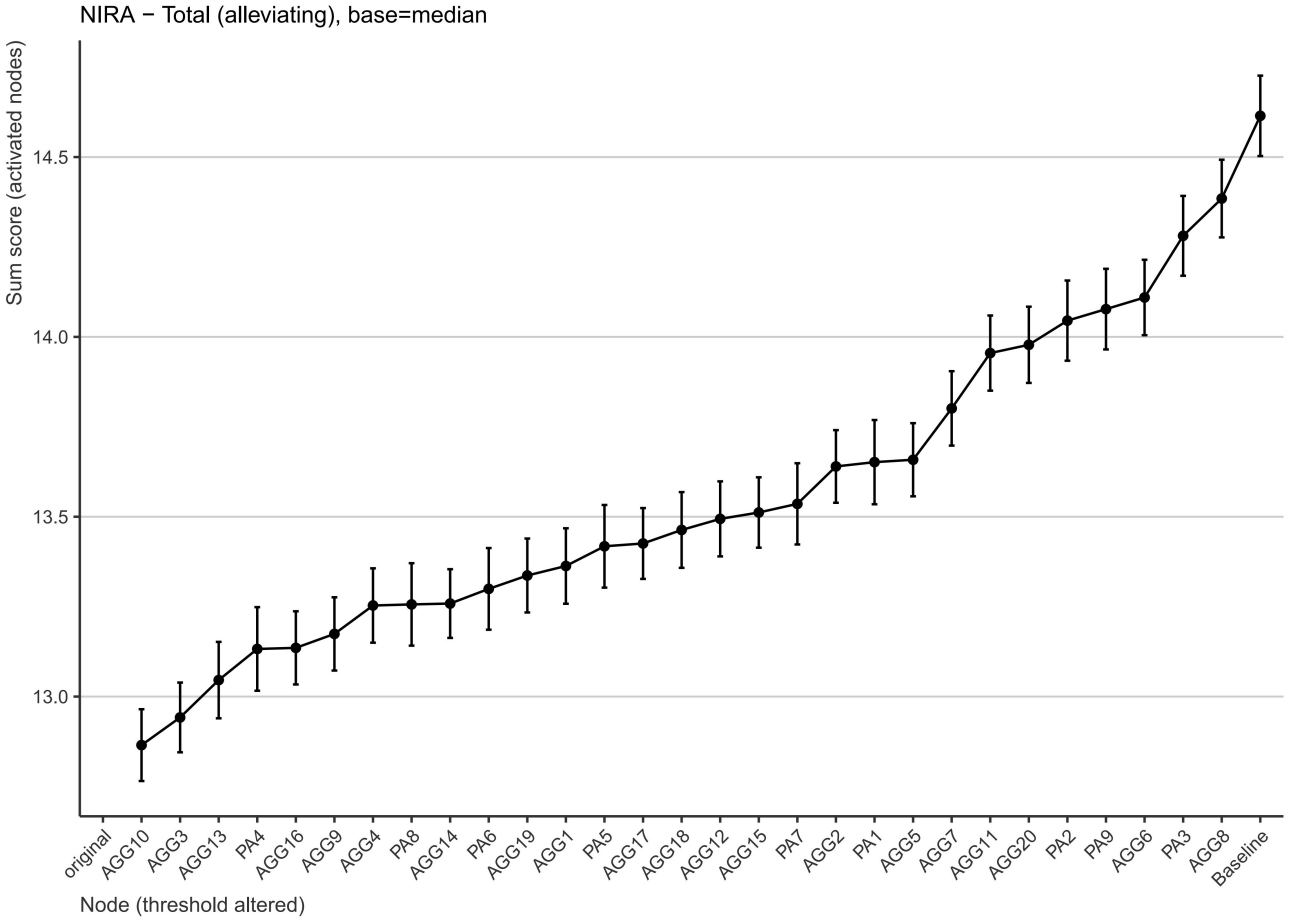
Network intervention analysis (NiRA) under the alleviating condition.

In the alleviating condition (Figure 3), changes in several PA- and AGG-related nodes were associated with lower overall network activation. This pattern suggests that both behavioral domains may be relevant to simulated decreases in system activation, although these results should be interpreted as model-based sensitivity patterns rather than evidence of intervention efficacy.

**Figure 4:**
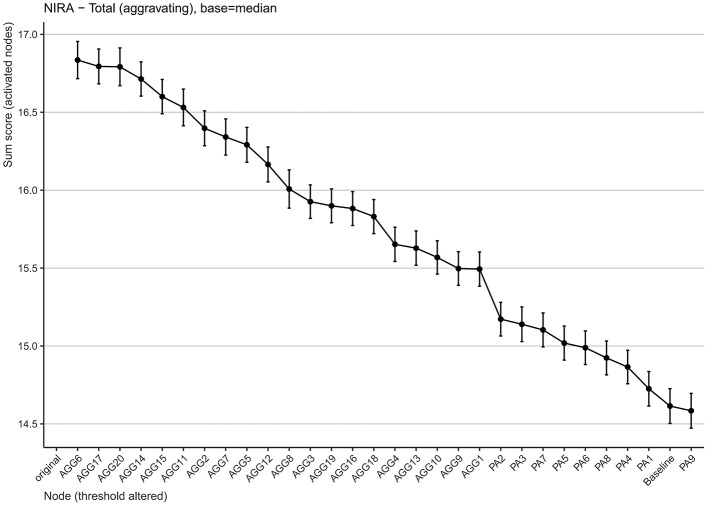
Network intervention analysis (NiRA) under the aggravating condition.

By contrast, in the aggravating condition (Figure 4), the nodes associated with the greatest increases in total network activation were mainly AGG items, whereas most PA items remained closer to the middle or lower part of the distribution. This pattern suggests that increases in overall network activation were more strongly linked to aggression-related components in the fitted model.

### 3.5 Bayesian graph results

**Figure 5:**
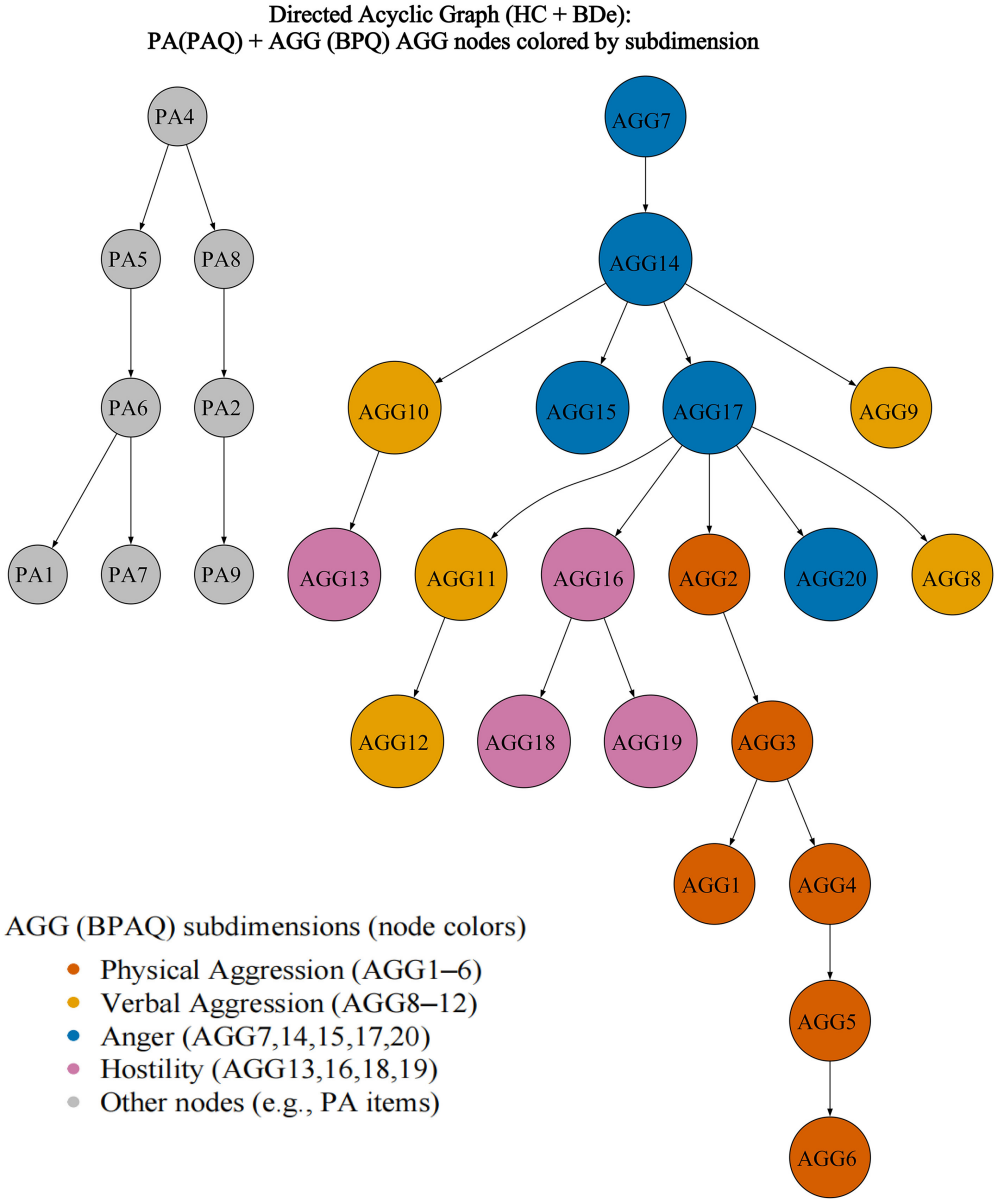
Bayesian directed acyclic graph (DAG) of physical activity (PA) and aggressive behavior (AGG) items. The graph was estimated using hill-climbing search with the Bayesian Dirichlet equivalent (BDe) score. Nodes represent individual PA and AGG items, and directed edges indicate probabilistic dependency structures learned from the data. AGG nodes are colored according to their theoretical subdimensions (physical aggression, verbal aggression, anger, and hostility), while PA items are shown in gray. Arrowheads indicate the orientation of probabilistic dependencies learned by the Bayesian algorithm and should not be interpreted as temporal order or causal effect.

A Bayesian directed acyclic graph (DAG) was estimated to examine asymmetric probabilistic dependency patterns among PA and AGG items. The resultant graph is shown in Figure 5, with AGG items color-coded by their theoretical subdimensions and PA items plotted separately.

The AGG domain displayed a more ordered and hierarchical dependency structure in the learned Bayesian graph. Several anger-related items occupied upstream positions in the DAG, including AGG14, which appeared as a parent node with multiple outgoing edges to AGG items from different subdimensions. These downstream nodes included other anger items (e.g., AGG15 and AGG17) as well as verbal-aggression and hostility items, indicating a dependency structure centered on anger-related content.

Directed edges also connected anger-related nodes with physical-aggression indicators. For example, AGG17 was directly linked to AGG2, which was in turn linked to AGG3. AGG3 then connected to AGG1 and AGG4, followed by AGG5 and AGG6. This organization is compatible with a hierarchical dependency pattern linking affective and behavioral components of aggression, but it should not be interpreted as evidence that anger temporally causes physical aggression.

PA items formed a relatively separate substructure in the Bayesian network. Directed edges among PA items mainly reflected internal dependencies among activity frequency, duration, and intensity indicators, with no PA node emerging as a strong upstream parent of AGG items. Similarly, edges from AGG items to PA items were sparse, indicating limited cross-domain dependency relationships in the learned graph.

Overall, the Bayesian graph analysis supported the general structure captured by the GGM by identifying asymmetric dependency patterns mainly within the AGG domain while also supporting the relative separation between PA and AGG systems. Because all analyses were based on cross-sectional data, these orientations should be treated as exploratory dependency patterns rather than causal pathways.

## 4 Discussion

The present study examined the undirected and directed network structure of physical activity and aggressive behavior among adolescents. The undirected GGM showed that PA and AGG items were largely organized into two relatively separate clusters, with stronger positive associations within each domain and weaker, less frequent associations between domains. Within the AGG cluster, the item-level structure broadly corresponded to the theoretical subdimensions of physical aggression, verbal aggression, anger, and hostility. Expected-influence analyses further indicated that anger-related items (AGG14, AGG17, and AGG15) and one physical-aggression item (AGG3) occupied the most influential positions in the network. NiRA suggested asymmetric activation patterns across alleviating and aggravating simulations, whereas the Bayesian DAG placed anger-related items in upstream positions within the learned probabilistic dependency structure. Taken together, these findings describe the component-level organization of PA and aggressive behavior, but they do not establish temporal sequence or causality.

The undirected model revealed dense within-domain associations in both PA and aggression. Within the aggression cluster, strong conditional associations between anger and physical-aggression items suggest that affective and behavioral components are closely interconnected after adjusting for other included nodes. This finding is consistent with the General Aggression Model and social cognitive accounts, which emphasize the joint involvement of affective arousal, hostile cognition, and behavioral expression in aggressive tendencies (Quan et al., 2019; Smith et al., 2026; Allen et al., 2018; Anderson and Bushman, 2002; Bandura, 1999). However, because the current data are cross-sectional, these associations should be interpreted as contemporaneous conditional dependencies rather than reciprocal processes unfolding over time.

The relatively sparse PA-AGG connections are also theoretically informative. Traditional variable-centered studies often report macro-level negative associations between physical activity and aggression, suggesting that higher activity levels may be associated with better psychosocial adjustment (Rhodes and Nigg, 2011; Spruit et al., 2016; Zhu et al., 2022). In contrast, the present item-level network suggests that PA and AGG components are not strongly connected through numerous direct item-to-item pathways. One interpretation is that the protective role of physical activity, if present, may operate through broader system-level processes such as emotion regulation, social participation, stress reduction, or school context rather than through direct suppression of specific aggression items. This interpretation remains hypothetical and should be tested in longitudinal or experimental designs.

The centrality findings highlight the structural importance of anger-related and physical-aggression items. AGG14, AGG17, AGG15, and AGG3 had high expected influence, suggesting that these items were broadly connected to other components of the observed aggression network. In network terms, central nodes may be useful for generating hypotheses about candidate intervention targets, but centrality alone does not prove that changing these nodes will causally change the wider system. Therefore, the present results should be used to prioritize future longitudinal monitoring and intervention trials rather than to make direct clinical claims.

The Bayesian DAG provided additional information about asymmetric probabilistic dependencies. In the learned graph, anger-related nodes, particularly AGG14, were positioned upstream of several other aggression components. This pattern is compatible with theories that emphasize anger and emotion regulation as important correlates of aggressive behavior (Berkowitz, 2012; Pop et al., 2025; Zhou and Zhen, 2022). Nevertheless, DAG orientation in cross-sectional data can be affected by distributional assumptions, omitted variables, and equivalent graph structures. Therefore, the DAG should be interpreted as an exploratory representation of conditional dependency orientation, not as evidence that anger precedes or causes subsequent aggressive behavior.

The separate PA substructure in the Bayesian graph further supports the relative independence of PA and AGG at the item level. Directed edges among PA items mainly reflected internal organization among activity indicators, whereas cross-domain dependencies were limited. This finding reinforces the need to distinguish between macro-level associations and item-level conditional dependencies. It also suggests that future research should examine possible mediating or moderating variables, such as emotion regulation, peer support, school climate, or family functioning, to clarify how physical activity may be connected to aggressive behavior.

## 5 Implications

The findings have implications for school-based assessment and hypothesis generation. Anger-related items and one physical-aggression item emerged as structurally influential components in the observed network, suggesting that anger regulation and aggressive behavioral expression may be useful domains for screening and preventive work. However, these implications should be framed cautiously: the present cross-sectional network identifies candidate components for further study rather than verified causal intervention targets.

For practice, school-based programs may consider combining physical-activity promotion with emotion-regulation education, conflict-resolution training, and psychoeducation about anger and hostile attribution (Ouyang and Liu, 2023; Smith et al., 2026). The sparse PA-AGG cross-domain structure suggests that physical activity alone may not directly alter specific aggression components unless it is embedded within broader psychosocial supports. For research, the present results provide a component-level map that can guide future longitudinal studies and intervention trials designed to test whether changes in anger-related components are followed by changes in physical or verbal aggression.

## 6 Strengths and limitations

This study contributes to the literature by applying both undirected GGM and Bayesian DAG approaches to the item-level relationship between physical activity and aggressive behavior among adolescents. The combination of expected influence, bootstrap stability evaluation, NiRA, and Bayesian graph analysis provided a fine-grained description of the PA-AGG component system. The relatively high correlation-stability coefficient for strength (CS = 0.75) supports the interpretability of the strength rankings.

Several limitations should be noted. First, the cross-sectional design precludes conclusions about causality or temporal precedence. Although the Bayesian DAG provides oriented dependency patterns, these orientations should be interpreted as exploratory and hypothesis-generating. Second, the data were based on self-report questionnaires, which may be affected by recall bias and social desirability, especially for aggressive behavior in school settings. Third, the anonymous field-survey procedure did not retain demographic information for students who declined participation or were absent, preventing a formal participant-vs.-non-participant comparison. Fourth, the final network analyses used complete PAQ-A and BPAQ item-level data and did not use imputation; therefore, the results apply to adolescents with complete responses on the analyzed nodes. Fifth, the network was estimated only with PA and AGG items. Because other relevant variables, such as emotion dysregulation, anxiety, depression, family functioning, and peer relationships, were not included, some edges may partly reflect omitted-variable effects. Sixth, the negative cross-domain PA-AGG edges retained by regularization should be interpreted as small conditional associations within the fitted model rather than individually confirmed causal or statistically significant effects. Future studies should use longitudinal, multi-informant, and covariate-enriched designs to test the robustness and temporal meaning of the observed dependency patterns. Although the available sociodemographic variables included sex, age, grade, only-child status, single-parent family status, and area of residence, direct indicators of socioeconomic status, such as household income, parental education, and parental occupation, were not collected. Consequently, the sample could not be further differentiated according to socioeconomic status.

## 7 Conclusion

This study used network and Bayesian graph methods to examine the item-level organization of physical activity and aggressive behavior among Chinese adolescents. The findings showed that aggression-related components, particularly anger-related items and one physical-aggression item, were central within the observed network, whereas PA and AGG items were relatively separate at the component level. The Bayesian DAG placed anger-related items in upstream positions within the learned probabilistic dependency structure, but these orientations should be understood as exploratory conditional dependencies rather than causal or temporal pathways. Overall, the study provides a component-level description of the PA-AGG system and offers empirically grounded hypotheses for future longitudinal and intervention research on adolescent aggression and physical activity.

## Data Availability

The original contributions presented in the study are included in the article/Supplementary material, further inquiries can be directed to the corresponding author.
